# Solvent mediated synthesis of multicolor narrow bandwidth emissive carbon quantum dots and their potential in white light emitting diodes

**DOI:** 10.1038/s41598-024-75476-0

**Published:** 2024-10-22

**Authors:** Mohamed Sami, Mohamed E. El-Khouly, Mohsen Ghali

**Affiliations:** 1 Energy Materials Program, Institute of Basic and Applied Sciences, Egypt-Japan University of Science and Technology, New Borg El-Arab City, Alexandria 21934 Egypt; 2 Nanoscience Program, Institute of Basic and Applied Sciences, Egypt, Japan University of Science and Technology, New Borg El-Arab City, Alexandria 21934 Egypt

**Keywords:** Carbon quantum dots, Narrow bandwidth emission, High fluorescence quantum yield, White light-emitting diodes, Materials science, Nanoscience and technology, Optics and photonics, Physics

## Abstract

The development of efficient and environmentally sustainable materials for white light-emitting diodes (WLEDs) is of paramount importance in the field of lighting technology. In this study, we present a solvent-modulated synthesis approach for the fabrication of multicolor narrow-bandwidth emissive carbon quantum dots (CQDs) as a promising solution for constructing WLEDs. The synthesis method involves the controlled reaction of organic precursors in different solvent environments, leading to the formation of CQDs with distinct emission wavelengths with a relatively small full width at half maximum, ranging from 28 to 42 nm. Moreover, these synthesized multicolor CQDs demonstrate a remarkably high fluorescence quantum yield of up to 65%, indicating their potential for constructing efficient WLED when incorporated in polymer matrix and coated on the surface of blue light-emitting diode (LED).

**Supplementary Information:**

The online version contains supplementary material available at 10.1038/s41598-024-75476-0.

## Introduction

Artificial lighting has played a significant role in human life and various industries over time. The progress of light sources, from fire to modern electric lights such as incandescent lamps, fluorescent tubes, and white light-emitting diodes, has been remarkable^1^. WLEDs have emerged as a particularly promising innovation in recent years because of their small size, uncomplicated design, extended lifespan, and adaptability for both indoor and outdoor lighting needs^2^. They are widely utilized for lighting and display purposes while also showing considerable promise in visible light technologies^3^. Three main methods are utilized in the development of WLEDs. The initial method involves placing a yellow emitting phosphor on blue LEDs, such as Y_3−x_Ce_x_Al_5_O_12_ (YAG: Ce) coated onto GaN blue chips. Another approach is to apply red, green, and blue phosphor layers on ultraviolet chips. Lastly, UV chips combined with white emission phosphor layers can also be used in the production of WLEDs^1^.

Moreover, different quantum dots (QDs) materials have been applied for achieving efficient WLEDs, including inorganic semiconductor quantum dots (QDs) such as high-quality InPZnS QDs^4^, CdSe/CdS/ZnS core/shell QDs^5^, InP/ZnS core/shell QDs^6^. Furthermore, huge effort has been exerted to develop perovskite quantum dots with ultra-high quantum yield for WLEDs development^7^. Although materials such as perovskites and inorganic QDs possess desirable optical properties like high fluorescence quantum yields, tunable wavelength emission, as well as narrow bandwidth emission, yet these materials are intrinsically toxic, unstable, environmentally destructive, costly ineffective, and complex to synthesize^8,9^.

Xu et al. uncovered fluorescent fragments of carbon nanotubes while purifying single-walled carbon nanotubes in 2004^10^. In 2006, Sun and colleagues produced fluorescent nanoscale carbon particles via laser ablation of a carbon target, which they called “carbon dots”^11^. As is known, carbon quantum dots (CQDs) are small spherical nanostructures made of carbon that measure less than 10 nm^12^. The surface of these dots can be modified with different chemical groups, making them suitable for a wide range of applications^13^. These carbon-based quantum dots have several advantages over traditional semiconductor quantum dots, including low toxicity, high photostability, and facile synthesis^14^. The recently identified CQDs surpass traditional metal and semiconductor quantum dots as well as organic dyes in various ways because of their minimal toxicity, compatibility with biological systems, high fluorescence efficiency, exceptional stability against photobleaching, and adjustable emission wavelength^15^.

Significant efforts have been dedicated to producing high-quality CQDs from various sources and improving their optical properties for use in multiple fields^16,17^. Basically, two main approaches were utilized to fabricate CQDs: top-down and bottom-up strategies^18^. These methods can be tailored to synthesize carbon quantum dots (CQDs) with specific band gaps, heteroatom doping, and functional groups^19,20^. Moreover, the structural properties of the precursor materials can be leveraged to define the characteristics of the resulting CQDs through the concept of structural memory^16^.

Furthermore, carbon quantum dots can be easily produced from a variety of carbon sources, including chemicals and biomasses, on a large scale^21,22^. Due to their unique characteristics, CQDs are widely utilized in numerous applications such as bioimaging^23^, drug delivery^24^, chemical sensing^25^, anti-counterfeiting measures^26^, photocatalysis^27^, solar cells^28^, WLEDs^29^, and more. Narrow-bandwidth quantum dots were employed to fabricate photoluminescence-based white light-emitting diodes, as they provide substantial benefits for white LED manufacturing. These quantum dots demonstrate high color selectivity, tunable emission characteristics, and enhanced quantum yield. Their narrow emission spectra enable the production of vibrant, saturated colors in contrast to broadband emissive phosphors. This results in a high color rendering index and enhances luminous efficacy by minimizing spectral overlap between emission bands^30,31^.

**Fig. 1:**
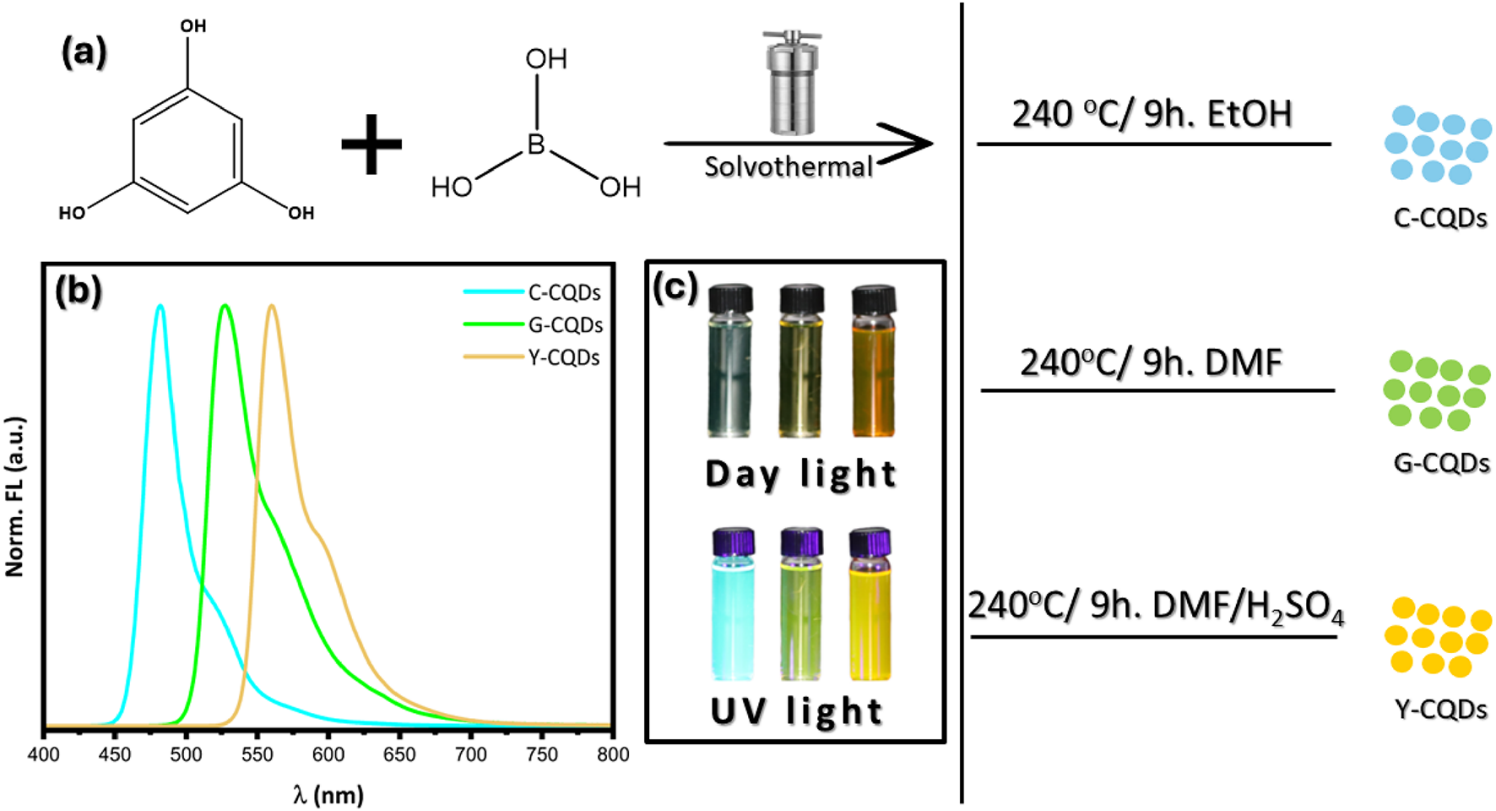
(**a**) Schematic diagram of the synthesis of multicolor carbon quantum dots. (**b**) combined fluorescence spectra of the multicolor CQDs samples. (**c**) Photographs of the C-CQDs, G-CQDs, and Y-CQDs dispersed in ethanol under daylight.

Based on the considerations above, we report the production of narrow bandwidth emissive cyan-carbon quantum dots (C-CQDs), green-carbon quantum dots (G-CQDs), and yellow-carbon quantum dots (Y-CQDs) through solvent-engineering of the solvothermal reaction involving three-fold symmetrical phloroglucinol and boric acid as depicted in Fig. (1a–c). The resulting multicolor carbon quantum dots were combined with polyvinylpyrrolidone and deposited on a blue light-emitting diode, culminating in the fabrication of a white light-emitting diode (WLEDs).

## Results and discussion

### Elemental and nanostructural analysis of multicolor CQDs

**Fig. 2:**
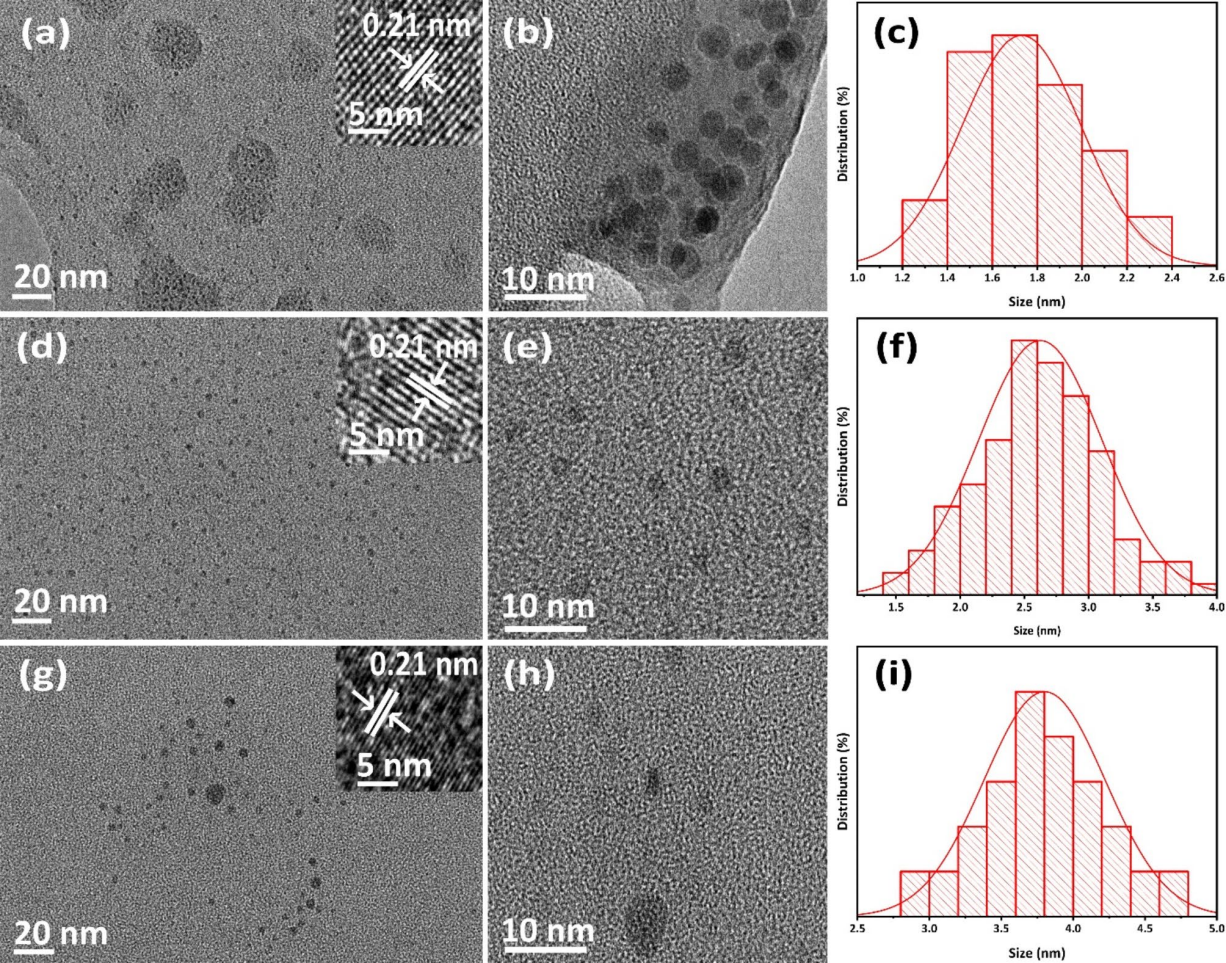
TEM, HR-TEM images, and particle size distribution of (**a–c**) C-CQDs, (**d–f**) G-CQDs, and (**g–i**) Y-CQDs.

The transmission electron microscope images presented in Fig. 2a-i provide detailed insights into the morphology and lattice spacing of the synthesized multicolor carbon quantum dots. Figure 2a showcases the spherical structure of the C-CQDs, with a lattice spacing of 0.21 nm, as highlighted in the inset. The uniformity of the C-CQDs is further corroborated in Fig. 2b. The semi-spherical morphology of the G-CQDs is illustrated in Fig. 2d, also exhibiting a lattice spacing of 0.21 nm, as shown in the inset, with Fig. 2e providing additional evidence for this observation. Similarly, Fig. 2g reveals that the Y-CQDs have a structure like both the C-CQDs and G-CQDs, with an interlayer spacing of 0.21 nm, which is confirmed in Fig. 2h. The corresponding size distributions of the C-CQDs, G-CQDs, and Y-CQDs are presented in Fig. 2c and f, and 2i, indicating average particle sizes of 1.7 nm, 2.6 nm, and 3.8 nm, respectively. Moreover, the supplementary TEM and high-resolution TEM images in Figure S1 further emphasize the amorphous spherical nature of these multicolor CQDs.

**Fig. 3:**
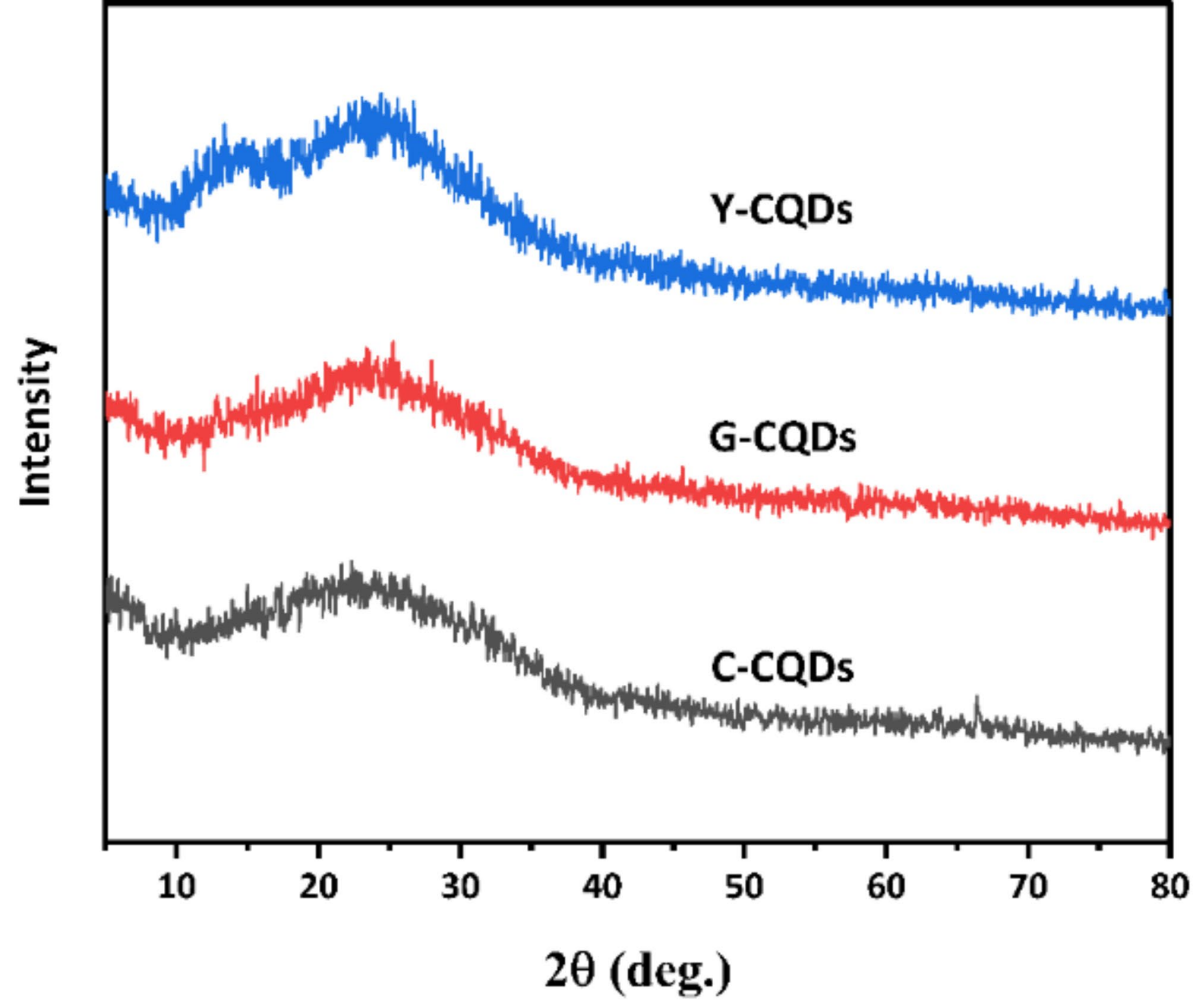
XRD patterns for the synthesized multicolor CQDs.

TEM analysis, along with X-ray diffraction spectroscopy, suggests that the C, G, and Y-CQDs produced are carbon-based structures with a semi-crystalline nature. Figure 3 exhibited a prominent broad XRD peak at 2q = 22.3° for all the samples corresponding to an interlayer lattice spacing of 0.398 nm, this value exceeds the crystallographic plane distance of bulk graphite (typically 0.33 nm) due to functional groups attached to the surface of multicolor CQDs^32^. Additionally, faint peaks at 2q = 42.9° indicate another interplanar distance of 0.21 nm, mostly attributed to specific planes; this demonstrates a certain level of crystallinity in the synthesized CQDs. Moreover, the observed XRD peak at 2q = 13.6° for Y-CQDs is often caused by their deformation resulting from the addition of sulfuric acid into the reaction precursors.

**Fig. 4:**
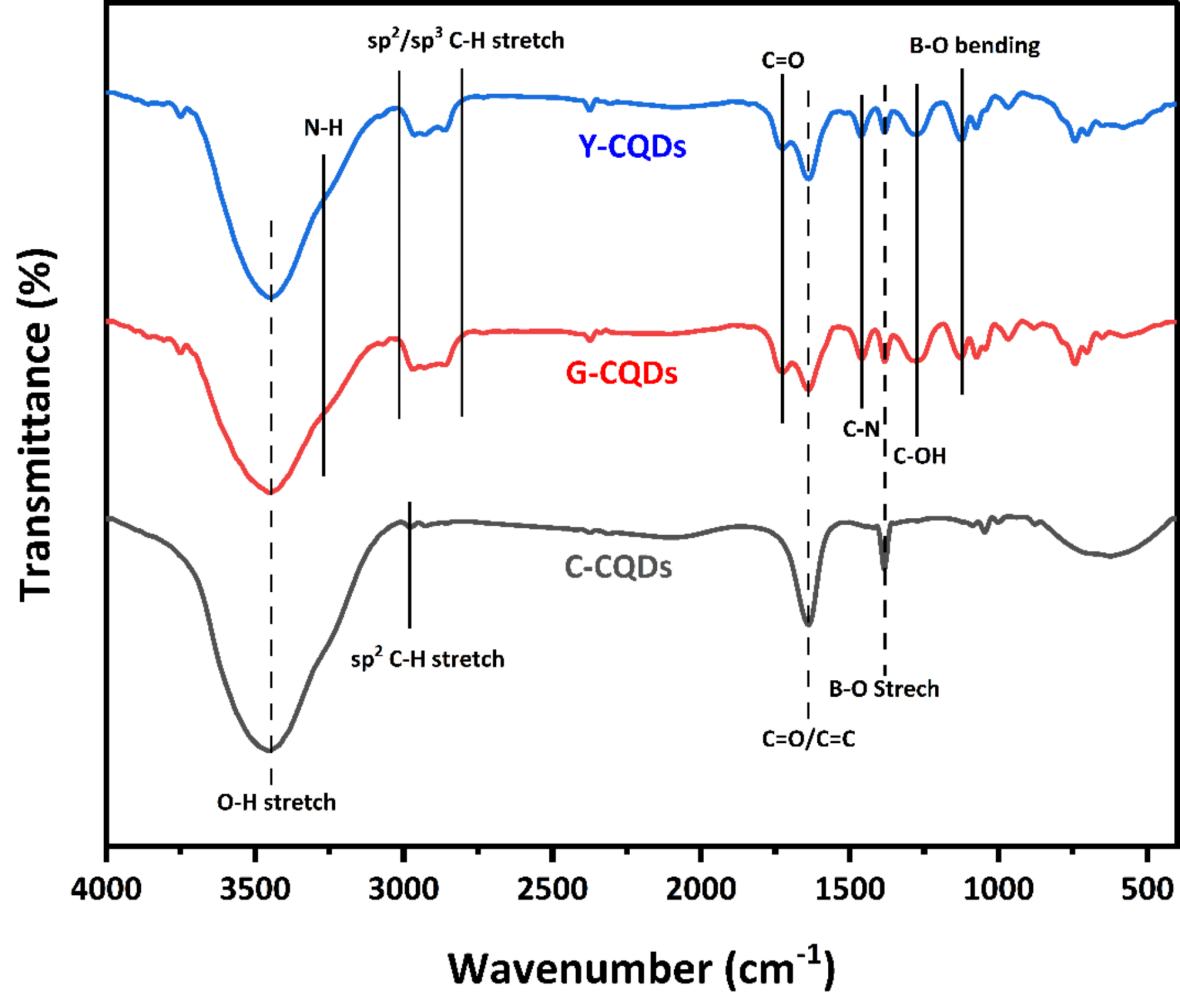
FT-IR spectra for the synthesized multicolor CQDs.

Fourier transform infrared analysis was used to examine the surface functional groups and bond structure of the C, G, and Y-CQDs that were synthesized. Figure 4 shows the IR transmission spectrum for the synthesized C-CQDs in the black line, while G-CQDs and Y-CQDs are illustrated in red and blue, respectively. For the C-CQDs, there is a broad peak around 3449 cm^−1^ that is ascribed to O-H stretching vibrations^33^. Additionally, a small peak was observed at 2980 cm^−1^ due to the sp^2^ C-H stretching^34^. The absorption peak at 1640 cm^−1^ was identified as vibrations of C = O/C = C^35,36^, while the absorption peak at 1385 cm^−1^ was attributed to B-O stretching vibrations^22^. In contrast, the G-CQDs and Y-CQDs have almost the same spectra. The recorded peaks at 3449 and 3260 cm^−1^ are attributed to the O-H stretching vibrations and the N-H bond^33^. A broad absorption peak in the region between 2990 and 2800 cm^−1^ was ascribed to the sp^2^/sp^3^ stretching vibration of the C-H bonds^34^. Additionally, the observed peaks around 1738, 1639, and 1457 cm^−1^ are mostly ascribed to C = O, C = C, and C-N, respectively^33,35,36^. Finally, the observed peaks at 1385, 1272, and 1133 cm^−1^ are attributed to B-O stretching vibrations, C-OH bending vibrations, and asymmetrical stretching of the oxygen atoms connected to the trigonal boron atoms^22,37^.

**Fig. 5:**
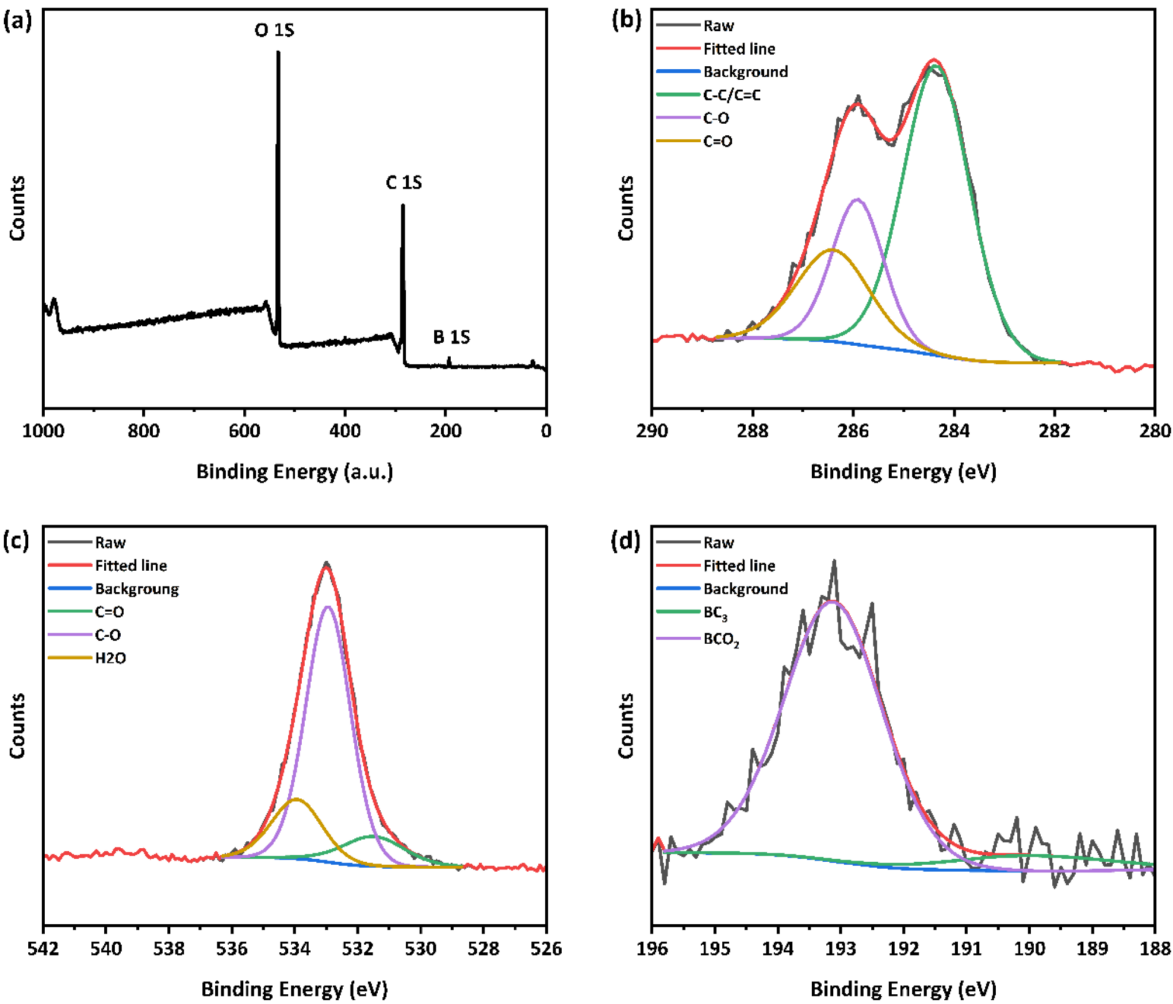
(**a**) XPS survey of C-CQDs. (**b**) XPS of C 1s, (**c**) O 1s and (d) B 1s.

Figure 5 presents the x-ray photoelectron spectroscopy (XPS) analysis for the synthesized C-CQDs. Figure 5a shows a survey spectrum of C-CQDs, indicating that these C-CQDs primarily consist of carbon, oxygen, and boron. Furthermore, Fig. 5b reveals that the high-resolution spectra of C 1s can be deconvoluted into three main peaks at 284.4, 285.9, and 286.4 eV, which can be attributed to C–C/C = C, C–O (hydroxyl), and C = O (carbonyl), respectively. In the O 1s Spectra, the peaks at 531.5 and 532.9 eV are assigned to the binding energies of C = O and C–O, respectively^38,39^, while the peak at 533.9 eV may be due to the presence of water molecules in the sample. The B 1s spectrum exhibits two peaks at 189.9 and 193.1 eV, which are ascribed to BC3 and BCO2^40^, as demonstrated in Fig. 5d.

The XPS analysis for both G-CQDs and Y-CQDs, as shown in Figure S2, revealed that they mainly consist of carbon, oxygen, boron, and nitrogen. This suggests that doping G-CQDs and Y-CQDs with nitrogen was achieved by carrying out the reaction in DMF as the reaction medium under relatively high pressure and temperature, which is noticed form the chemical composition analysis that carried out by XPS and FT-IR techniques. The role of sulfuric acid in the preparation of the Y-CQDs was aimed to act as a catalysis to facilitate the carbonization prosses, as well as increase the size of the CQDs to be able to decrease the optical bandgap as seen in TEM images.

The atomic ratios presented in Table 1 reveal that the C-CQDs possess the lowest carbon content and the highest oxygen and boron contents, without the presence of any other chemical elements. Conversely, the G-CQDs demonstrate an increased carbon content coupled with a notable decrease in oxygen and boron contents, in addition to a significant nitrogen dopant. Lastly, the synthesized Y-CQDs exhibit the highest carbon content and the lowest oxygen and boron contents among the three samples, along with a substantial decrease in the nitrogen dopant compared to the G-CQDs.

**Table 1:** Elemental analysis of the multicolor CQDs based on XPS survey spectra.

	C (%)	O (%)	B (%)	*N* (%)
C-CQDs	64.34	33.89	1.77	---
G-CQDs	73.25	18.66	0.52	7.58
Y-CQDs	81.19	17.22	0.42	1.18

The high-resolution XPS data for the G-CQDs, as shown in Figure S3a, reveals that the C 1s spectra could be deconvoluted into three distinct peaks at binding energies of 283.9 (C-C), 285.5 (sp3 C-N), and 288 eV (C = O)^41−43^. O 1s band showed in Figure S3b was also deconvoluted into two peaks at 530.2 eV and 532.4 eV, which were attributed to C-O and C-OH bonds, respectively^44,45^. Furthermore, Figure S3c depicts the N 1s spectrum indicated the presence of a pyridine-type nitrogen structure at 399.1 eV^46^. B 1s spectrum illustrated in Figure S3d demonstrates the existence of a BCO_2_ bonding environment at 191.3 eV^47^. Similarly, high-resolution XPS analysis of the Y-CQDs presented in Figure S4a shows that the C 1s spectra could be deconvoluted into three distinctive peaks at 284.4, 285.2, and 285.9 eV. These peaks were attributed to C-C/C = C, C-O, and C-N/C-O bonding environments, respectively^48–50^. Furthermore, O 1s spectra Figure S4b exhibited three peaks with binding energies of 531.3 eV for C = O, 532.7 eV for C-OH, and 533.4 eV for C-O-^51–53^. Figure S4c shows that N 1s spectrum indicated the presence of an amide-type nitrogen structure at 399.8 eV^46^. The B 1s band presented in Figure S4d demonstrated the existence of a B-O bonding configuration at 192.3 eV^52^.

## Optical properties of multicolor CQDs

**Fig. 6:**
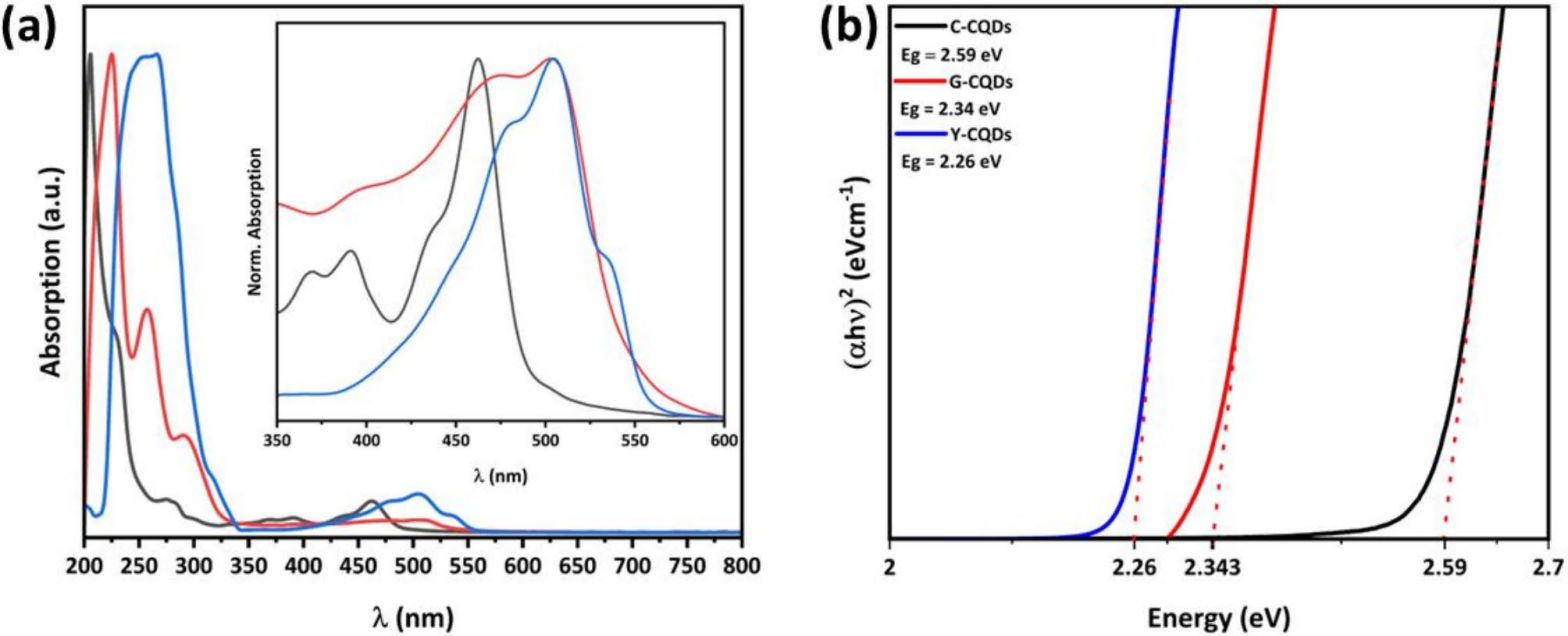
(**a**) Absorption spectra of C-CQDs (black line), G-CQDs (red line), and Y-CQDs (blue line). (**b**) The corresponding Tauc-plots.

The UV − visible absorption spectra of the synthesized CQDs illustrated in Fig. 6a exhibited similar absorbance in the UV range (200–350 nm) and varying absorbance at longer wavelengths, with absorption edges observed at 462, 505, and 535 nm for C-CQDs, G-CQDs, and Y-CQDs, respectively. Intense peaks of absorption in the ultraviolet range indicate the existence of electronic transitions from π to π* orbitals, largely associated with the conjugated C = C/C = O domain^54^. Furthermore, the change in absorption boundaries of the synthesized CQDs due to n → π* transitions is a consequence of size increase from 1.7 nm to 3.8 nm, emphasizing the impact of quantum confinement on these CQD samples^55^. In addition to the UV–Vis spectra, Tauc-Plot curves presented in Fig. 6b yield information on the calculated optical bandgap for these CQDs samples, revealing values of 2.59 eV for C-CQDs, 2.34 eV for G-CQDs, and 2.26 eV for Y-CQDs.

**Fig. 7:**
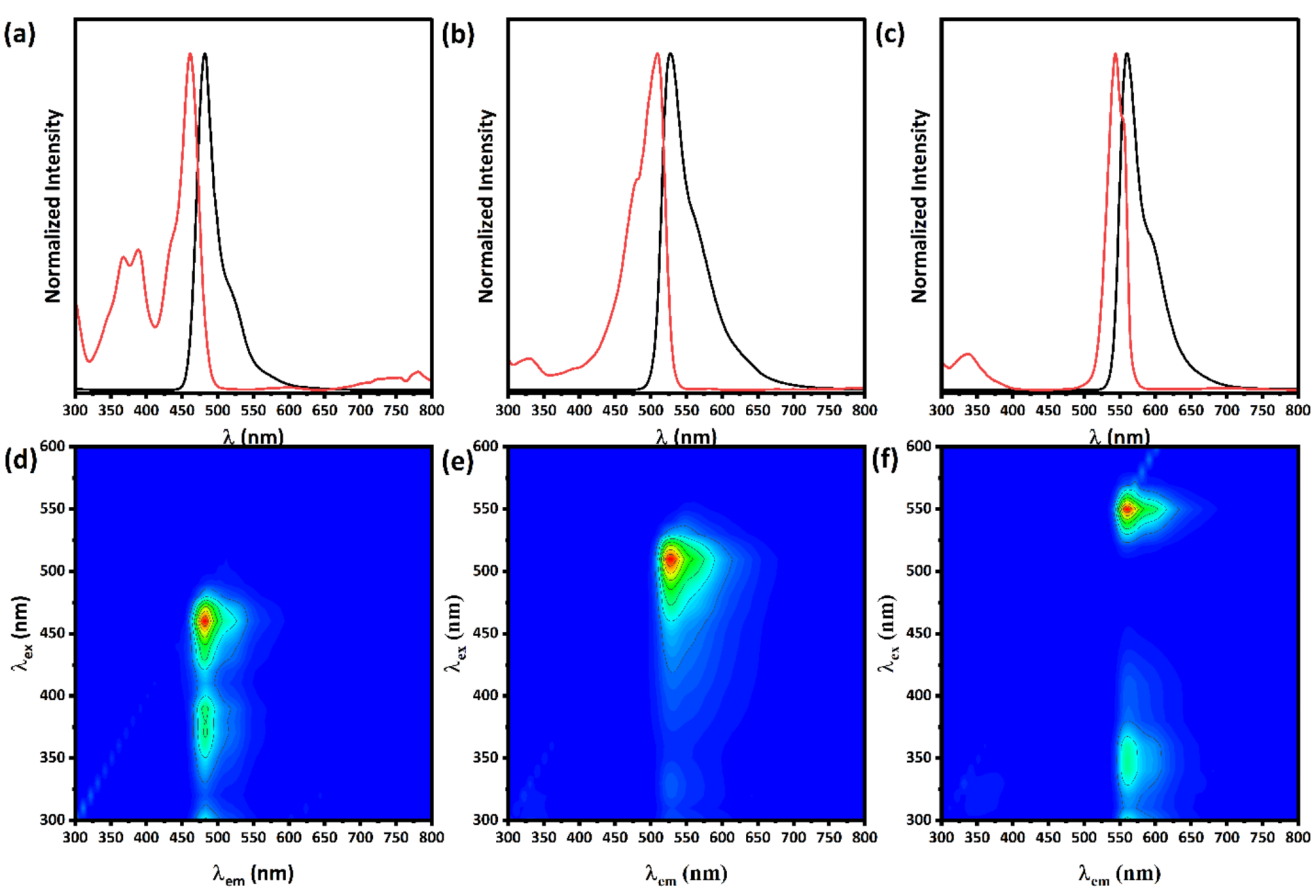
(**a**, **b**, and **c**) Fluorescence emission (black line) and fluorescence excitation (red line) spectra of the C-, G-, Y-CQDs. (**d**, **e**, and **f**) depicts the corresponding 2D excitation-emission maps.

Figure 7 illustrates the photoluminescence (PL) spectra of the synthesized CQDs with emission maxima at 482 nm, 527 nm, and 560 nm for the synthesized C-CQDs, G-CQDs, and Y-CQDs, respectively. The emissions of the CQDs samples are most notably characterized by their narrow bandwidth emissions, with full-width at half-maximum (FWHM) measuring 28 nm for C-CQDs, 42 nm for G-CQDs, and 33 nm for Y-CQDs. The presence of weak shoulder peaks at longer emission wavelengths in the photoluminescence spectra is primarily attributed to the excimer emission characteristics of the synthesized multicolor carbon quantum dots. Interestingly, as the concentration of the carbon quantum dots was increased, the intensity of the shoulder peak at the longer emission wavelengths also increased, as shown in Figure S6, indicating enhanced excimer-dimer interactions within the multicolor carbon quantum dot samples. Throughout the study, the multicolor carbon quantum dot samples were generally maintained at a concentration of 50 µg/mL.

Moreover, the photoluminescence excitation of multicolor CQDs depicted in Fig. 7 (a–c), which is perfectly match with the absorption spectra of these multicolor CQDs samples seen in the inset Fig. 6; along with the significantly small FWHM values suggest high color purity due to the direct exciton recombination, indicating their potential application in display technology and other photonic applications. Furthermore, both C-CQDs and G-CQDs show a small Stokes shift between photoluminescence excitation and emission of approximately 20 nm while Y-CQD exhibits only 13 nm shift, which are mostly attributed to band edge direct exciton recombination as well as weak electron–phonon coupling in the multicolor CQDs^56,57^. Moreover, Fig. 7(d–f) illustrate a two-dimensional excitation emission map that confirms the intrinsic narrow bandwidth characteristics of the synthesized multicolor CQDs samples as well as demonstrate excitations-independent nature of the synthesized C-, G-, Y-CQDs. By using fluorescein as the fluorescence quantum yield (FLQY)reference solution, the FLQY of the examined C-, G-, Y-CQDs were found to be 65%, 54%, and 60%, respectively. Figure S5 illustrates the remarkably stable photoluminescent properties of the synthesized multicolor carbon quantum dots, which were evaluated over a ten-hour time frame. All three types of CQDs, namely C-CQDs, G-CQDs, and Y-CQDs, displayed exceptional emission stability, maintaining nearly complete retention of their photoluminescent intensity throughout the observation period. Furthermore, Table S1 shows that the synthesized multicolor CQDs outperform many other reported CQDs in terms of quantum yield, and it also notes that they exhibit remarkably small Stokes shifts in comparison to others.

**Fig. 8:**
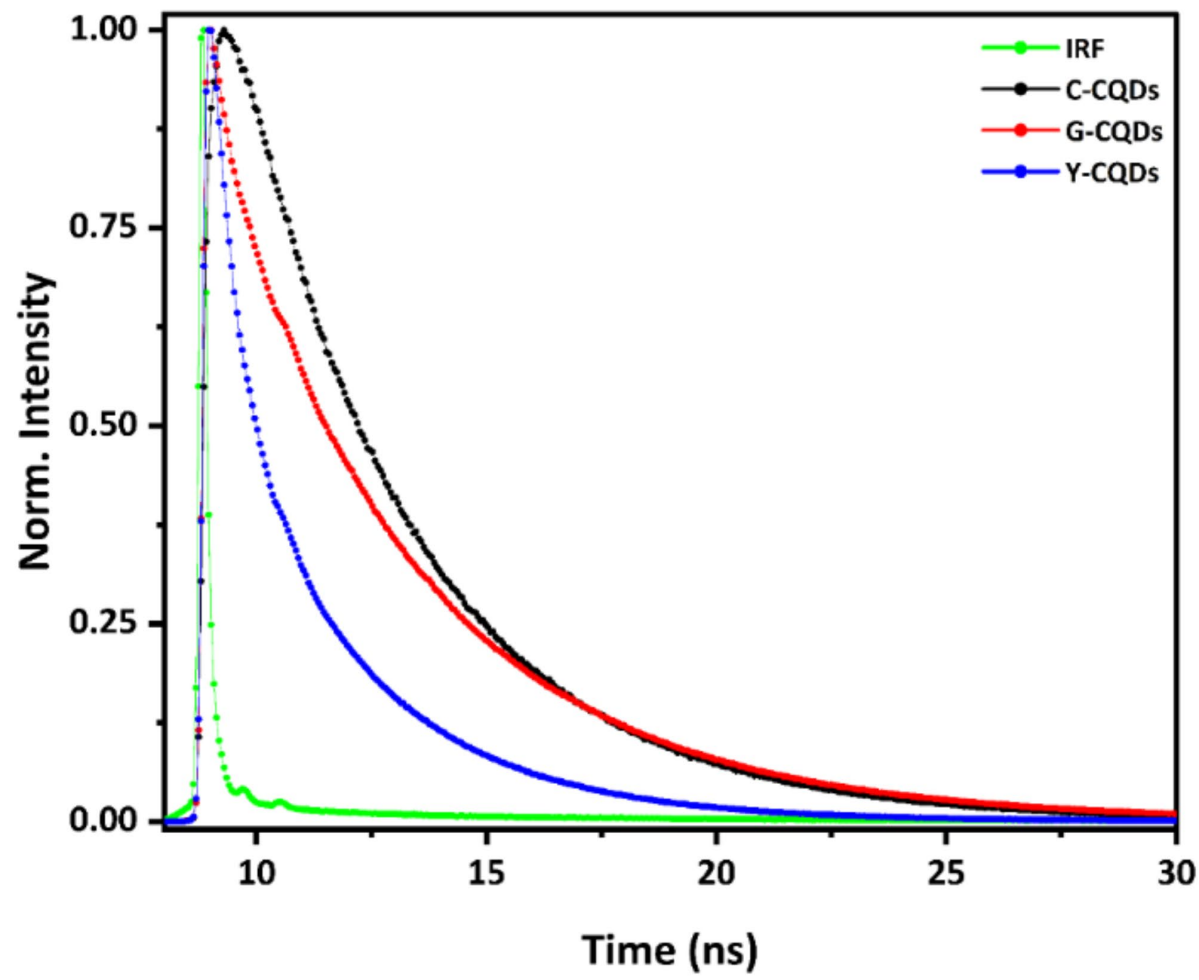
Fluorescence decay of the synthesized multicolor CQDs.

Moreover, exciton recombination dynamics by using time resolved PL spectra of our synthesized multicolor CQDs samples. The 439 nm picosecond pulsed laser was used to pump the CQDs samples and observe fluorescence lifetime decay at 482 nm, 527 nm, and 560 nm for C-, G-, and Y-CQDs respectively. Figure 8 display a single exponential fluorescence lifetime decay of almost 4.39 ns for C-CQDs solution indicates stable excitons with mainly radiative decay and minimal nonradiative contribution. Unlike C-CQDs, the fluorescence decay profiles of G-CQDs and Y-CQDs showed different features. As seen from Fig. 8, the decay profile of G-CQDs exhibited biexponential decays; where the fast decay was found to be 0.83 ns, and slow decay of 3.25 ns. Similarly, the fluorescence decay of Y-CQDs exhibited biexponential decay; the fast decay was found to be 0.52 ns and 3.80 ns. The differences in the fluorescence decay profiles could be caused by the presence of defect states introduced by the DMF synthetic solvent for both G-CQDS and Y-CQDs. The fluorescence lifetime of both G-CQDs and Y-CQDs have been fitted according to Eq. (1)

1 $$\:I\left(t\right)={I}_{0}+{B}_{1}{e}^{(-\frac{t}{{\tau\:}_{1}})}+{B}_{2}{e}^{(-\frac{t}{{\tau\:}_{2}})}$$

Where $\:I\left(t\right)$ is the fluorescence intensity as a function of time, $\:{I}_{0}$ is the initial fluorescence intensity, $\:t$ normalized to the intensity at $\:t=0$, $\:{\tau\:}_{1}$and $\:{\tau\:}_{2}$ are the fluorescence decay for the first and second decay components, $\:{B}_{1}$and $\:{B}_{2}$ are the fractional intensity of the first and second decay components.

The intensity average lifetime depicted in Table 2 has been calculated for both G-CQDs and Y-CQDs according to Eq. (2)^58^.

2 $$\:\langle{\tau\rangle}_{int}=\:\frac{\sum\:_{i=1}^{2}{B}_{i}{{\tau\:}_{i}}^{2}}{\sum\:_{i=1}^{2}{B}_{i}{\tau\:}_{i}}=\frac{{B}_{1}{{\tau\:}_{1}}^{2}+{B}_{2}{{\tau\:}_{2}}^{2}}{{B}_{1}{\tau\:}_{1}+{B}_{2}{\tau\:}_{2}}$$

**Table 2:** Fluorescence decay profiles for G-CQDs and Y-CQDs.

	$\:{\tau\:}_{1}\:\left(sec\right)$	$\:{\tau\:}_{2}\left(sec\right)$	$\:{B}_{1}$	$\:{B}_{2}$	$\:{\langle{\tau}}\rangle_{int}$
C-CQDs	$\:4.39\:\times\:{10}^{-9}$	---	$\:100$	---	---
G-CQDs	$\:8.26\times\:{10}^{-10}$	$\:3.249\times\:{10}^{-9}$	$\:20$	$\:80$	$\:3.104\times\:{10}^{-9}$
Y-CQDs	$\:5.22\times\:{10}^{-10}$	$\:3.80\times\:{10}^{-9}$	$\:58$	$\:42$	$\:3.277\times\:{10}^{-9}$

## Characterization of white light-emitting diode based on multicolor CQDs

**Fig. 9:**
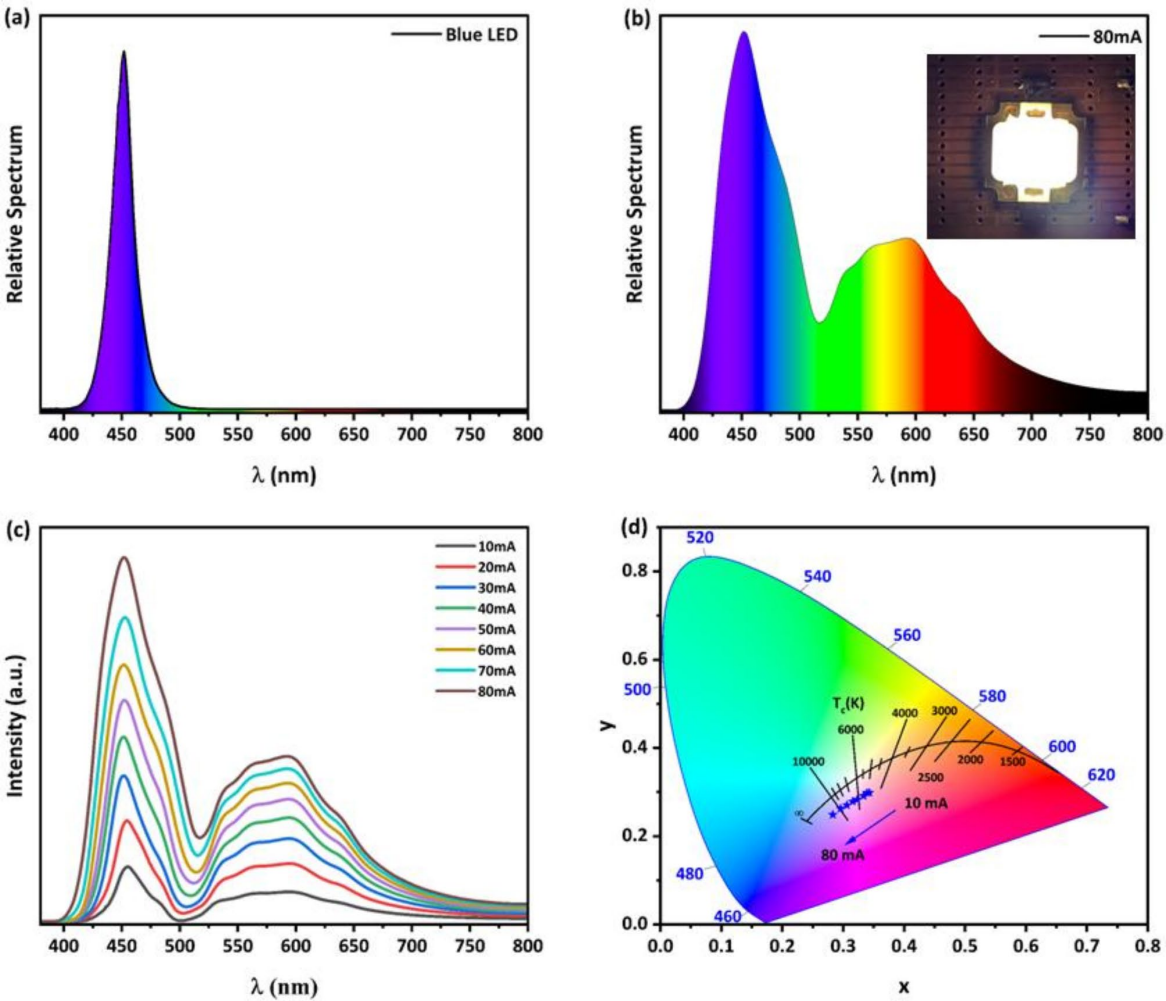
(**a**) Electroluminescence spectrum of blue LED. (**b**) Electroluminescence of the fabricated WLED. (**c**) WLED electroluminescence as a function of applied current. (**d**) corresponding CIE 1931 diagram.

The high fluorescence quantum yield of the synthesized multicolor C-, G-, and Y-CQDs allowed them to be combined with blue LED chips to create WLEDs. Initially, the C-, G-, and Y-CQDs were dispersed in ethanol and mixed in various proportions to measure the resulting emission using a Spectrofluorophotometer. This process yielded a white emitting solution of multicolor CQDs. Subsequently, PVP was added to the mixed solution to produce a 15 wt% multicolor CQDs solution in PVP. The blue light absorption capabilities of the individual layers were determined by recording the transmission spectra. The results showed that the C, G, and Y-CQD films can attenuate approximately 22.5%, 43.5%, and 62.4% of the 450 nm blue emission, respectively. See Figure S7 for details. A photo-driven white light-emitting diode based on fluorescent multicolor CQDs was then successfully assembled by applying a thick layer of the prepared PVP mixed solution onto the surface of a flat blue LED. Figure 9a shows an electroluminescence (EL) spectrum of blue LED chip with an emission peak at 450 nm, while Fig. 9b demonstrates that after encapsulating the blue LED with a fluorescent mixture of multicolor CQDs, it generated a white EL spectrum. The luminous intensity increased as the induced current increased, without changing the emission spectrum shape, as indicated by Fig. 9c. Additionally, the prepared WLED depicted in the inset of Fig. 9b showed a color rendering index CRI of 79% and correlated color temperature CCT of 5723 K and color coordinates (0.33, 0.30) when applied current is at 30 mA which corresponds to neutral white light suitable for indoor lighting. Furthermore, Fig. 9d showcases a shift from neutral white to cool white illumination as the applied current is increased.

**Fig. 10:**
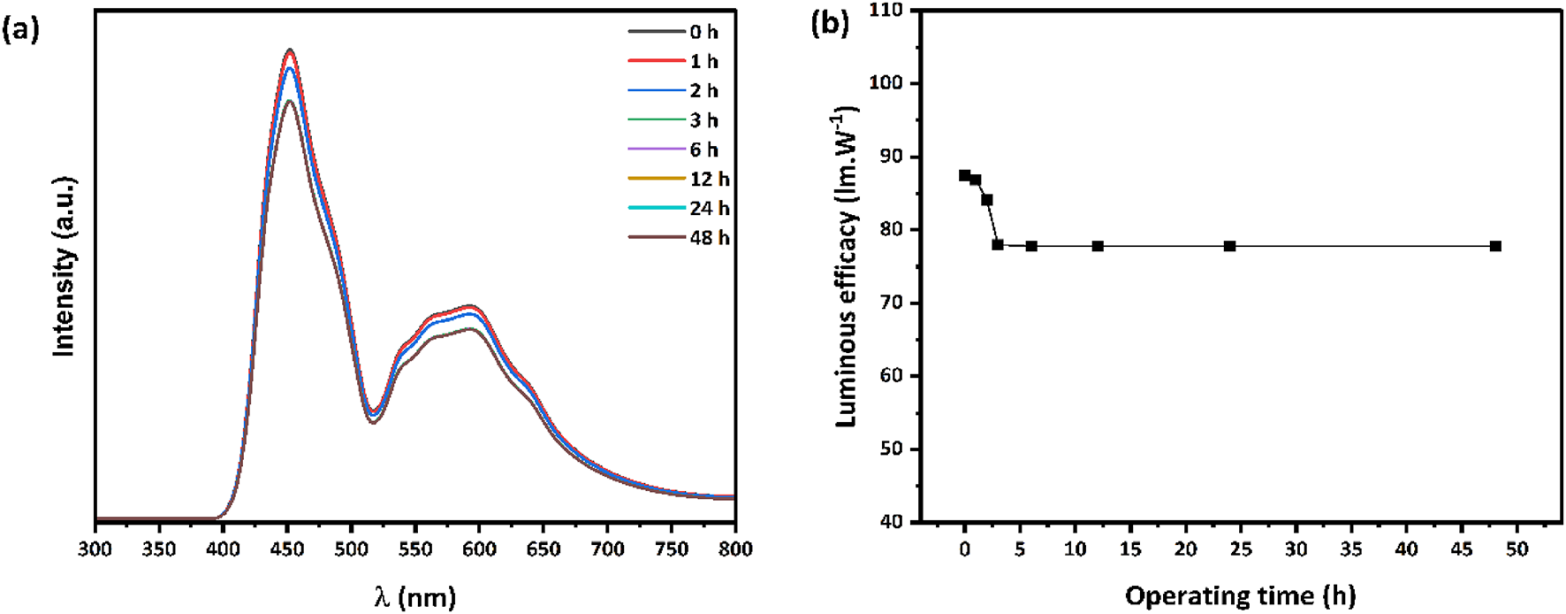
(**a**) EL spectrum of the WLEDs at different operating time intervals, (**b**) Luminous efficacy of the WLED as a function of the operating time.

Leveraging the high quantum yield of the synthesized multicolor carbon quantum dots, the optimized white LED device demonstrated a luminous efficacy of 87.5 lm/W when driven at 80 mA. However, this luminous efficacy decreased to 77.8 lm/W after 48 h of operation. Figure 10a presents the electroluminescence spectra of the fabricated WLED device measured over different operating time intervals, while Fig. 10b illustrates the luminous efficacy as a function of the operating time.

## Experimental details

### Materials

Phloroglucinol, boric acid, sulfuric acid, and polyvinylpyrrolidone (PVP) were purchased from Sigma Aldrich. Ethanol, dimethylformamide, methanol, dichloromethane, petroleum ether, ethyl acetate, and silica gel 150 A were obtained from Ficher chemicals. All chemicals were used without any purification. Deionized water was produced using an Elga ultrapure water system.

### Synthesis of cyan emissive carbon quantum dots (C–CQDs)

Narrow bandwidth cyan-emitting carbon quantum dots (C-CQDs) were synthesized via a solvothermal treatment of 1:1 molar ratio of phloroglucinol and boric acid. In detail, 400 mg of phloroglucinol and 196.11 mg of boric acid were dissolved in 10 mL of ethanol through sonication for 30 min. The resulting solution was then transferred into a 25 mL polypropylene-lined stainless-steel autoclave and heated at 240 °C for 9 h. The obtained solution was concentrated using a rotary evaporator and purified through silica column chromatography, with an increasing volume ratio of methanol in dichloromethane from 13:1 to 8:1. The purified carbon quantum dots dissolved in the dichloromethane/methanol mixture were then dried via rotary evaporation and redispersed in ethanol for further characterization.

## Synthesis of green emissive carbon quantum dots (G–CQDs)

Green carbon quantum dots (G-CQDs) with narrow bandwidth emission were synthesized via a solvothermal method. Specifically, 400 mg of phloroglucinol and 196.11 mg of boric acid were dissolved in dimethylformamide (DMF) and subjected to ultrasonic treatment for 30 min. The resulting solution was then transferred into a 25 mL polypropylene-lined stainless-steel autoclave reactor and heated at 240 °C for 9 h. After cooling down to room temperature, the obtained solution was precipitated using a 1:1 mixture of petroleum ether and ethyl acetate. The precipitated G-CQDs were dispersed in ethanol and further purified through silica column chromatography, employing an increasing volume ratio of methanol in dichloromethane from 18:1 to 10:1. The purified green carbon quantum dots in the dichloromethane/methanol mixture were dried and redispersed in ethanol for subsequent characterization.

## Synthesis of yellow emissive carbon quantum dots (Y–CQDs)

The production of yellow emissive narrow bandwidth emission involves the dissolving of 400 mg of phloroglucinol and 196.11 mg of boric acid into a mixture of 8 mL DMF and 2 mL of concentrated sulfuric acid with the aid of ultrasound waves. The resultant solution was heated at 240 °C for 9 h inside a 25 mL polypropylene-lined stainless-steel autoclave reactor. The obtained solution was precipitated using a 3:1 mixture of petroleum ether and ethyl acetate. The precipitated Y-CQDs were dispersed in ethanol and further purified through silica column chromatography, employing an increasing volume ratio of methanol in dichloromethane from 22:1 to 12:1. The purified yellow carbon quantum dots in the dichloromethane/methanol mixture were dried and redispersed in ethanol for subsequent characterization.

### Fluorescence quantum yield measurements

The fluorescence quantum yields (QY) of the examined C-, G-, Y-CQDs were determined through a relative quantum yield measurement using Shimadzu RF-6000 spectrofluorometer equipment with a 100 mm Spectralon integrating sphere and Shimadzu UV-2600 UV/Vis spectrophotometer according to the flowing equation:

3 $$\:{\varphi}_{x}={\varphi}_{st}\:.\:\left(\frac{{A}_{st}}{{A}_{x}}\right).\left(\frac{{F}_{x}}{{F}_{st}}\right).\:\left(\frac{{\eta\:}_{x}^{2}}{{\eta\:}_{st}^{2}}\right).\left(\frac{{D}_{x}}{{D}_{st}}\right)$$

In Eq. 3, the suffix *st* represents the standard sample and x represents the unknown sample. $\:\varphi$ is the quantum yield value, $\:A$ is the measured absorbance using UV-VIS spectrophotometer, $\:F$ is the are of the FWHM of the corrected emission spectrum peak, $\:\eta\:$ is the solvent refractive index, and $\:D$ is the dilution ratio of the sample during emission spectrum measurement compared to its concentration during UV-VIS spectrum measurement. Fluorescein was used as a quantum yield reference with QY value of 0.79.

### Fabrication of WLEDs based on multicolor CQDs

The produced C-CQDs, G-CQDs, and Y-CQDs solutions were combined using a mixing ratio of about 1:2:1.15, resulting in white emission when excited by blue radiation. PVP was then dispersed in the CQD mixture to formulate a 15 wt% CQDs@PVP solution, which was applied onto a flat blue LED chip to fabricate WLEDs.

### Characterization

Transmission electron microscopy (TEM) was carried out using a JEOL JEM-2100 transmission electron microscope, with an accelerating voltage of 200 kV. Fourier-transform infrared (FT-IR) spectra were recorded with a Bruker Invenio S Fourier FT-IR spectrometer. X-ray diffraction (XRD) patterns were conducted using Shimadzu Lab X 6100. XPS spectra were carried out using Thermo Fisher Scientific K-Alph X-ray photoelectron spectrometer. UV–VIS absorption spectra were recorded using Shimadzu UV-2600 spectrophotometer. The fluorescence quantum yields (QY) of the examined C-, G-, Y-CQDs were recorded by using Shimadzu RF-6000 spectrofluorometer equipment with a 100 mm Spectralon integrating sphere. Photoluminescence measurements were made using Shimadzu RF-6000 fluorescence spectrometer and fluorescence decay curves were constructed with Time Correlated Single Photon Counting (TCSPC, Horiba) spectrophotometer. WLEDs spectra were recorded using a LISUN group LMS-9000 C spectrometer.

## Conclusion

In conclusion, we reported the synthesis of cyan, green, and yellow emitting carbon quantum dots through solvent modulation of the solvothermal reaction. The obtained multicolor CQDs were characterized by a narrow full width at half maximum of approximately 28 nm and a high quantum yield up to 65%. These CQDs with narrow emission bandwidths demonstrate the capability to generate white light when combined with polyvinylpyrrolidone and deposited on a blue LED surface. The fabricated WLED exhibited a desirable color coordinate of (0.33, 0.30) with a correlated color temperature of 5723 K and a color rendering index of 79. The results indicate that the WLED based on multicolor CQDs has promising potential for indoor lighting applications.

## Electronic supplementary material

Below is the link to the electronic supplementary material.

Supplementary Material 1

## Data Availability

The datasets used and/or analyzed during the current study are available from the corresponding authors upon reasonable request.
