# Supplementary material for: Solvent mediated synthesis of multicolor narrow bandwidth emissive carbon quantum dots and their potential in white light emitting diodes
Source: Sci Rep. 2024 Oct 22;14:24812. doi: 10.1038/s41598-024-75476-0 (PMC11496623; doi:10.1038/s41598-024-75476-0)
Supplement: Supplementary file 1 — Supplementary Material 1 [file 41598_2024_75476_MOESM1_ESM.docx]

**Supporting Information**

Solvent-mediated synthesis of multicolor narrow bandwidth emissive carbon quantum dots and their potential in white light-emitting diodes

Mohamed Sami^1, *^, Mohamed E. El-Khouly^2^, and Mohsen Ghali^1, *^

^1^ Energy Materials Program, Institute of Basic and Applied Sciences, Egypt-Japan University of Science and Technology, New Borg El-Arab City, Alexandria, 21934, Egypt

^2^ Nanoscience Program, Institute of Basic and Applied Sciences, Egypt-Japan University of Science and Technology, New Borg El-Arab City, Alexandria, 21934, Egypt

| Corresponding authors: | [Mohamed.Sami@ejust.edu.eg](mailto:Mohamed.Sami@ejust.edu.eg) |
| --- | --- |
|  | Mohsen.Ghali@ejust.edu.eg |

Figure D1 presents TEM and HR-TEM images of the C-CQDs, G-CQDs, and Y-CQDs, further emphasizing the amorphous spherical nature of these multicolor carbon quantum dots.


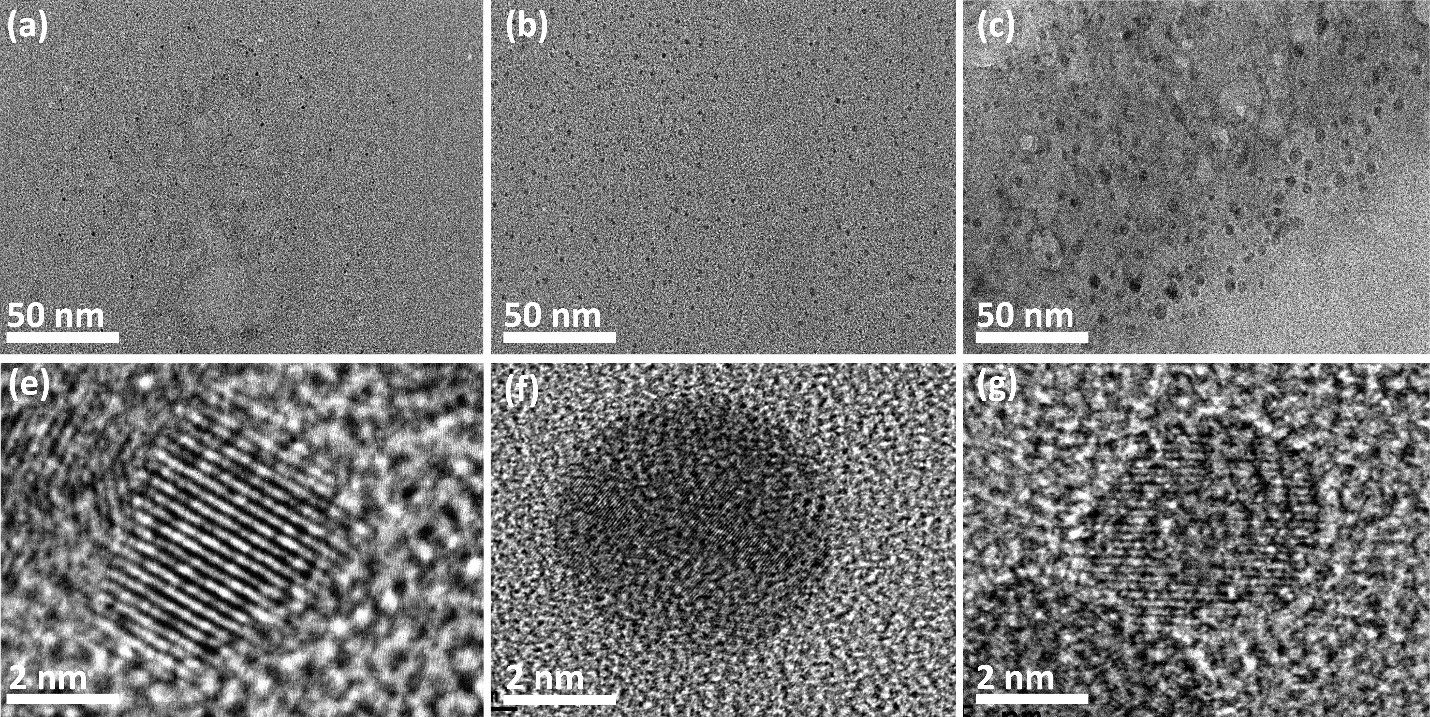


*Figure S1 shows the TEM and HR-TEM images of (a, e) C-CQDs, (b, f) G-CQDs, and (c, g) Y-CQDs.*

**
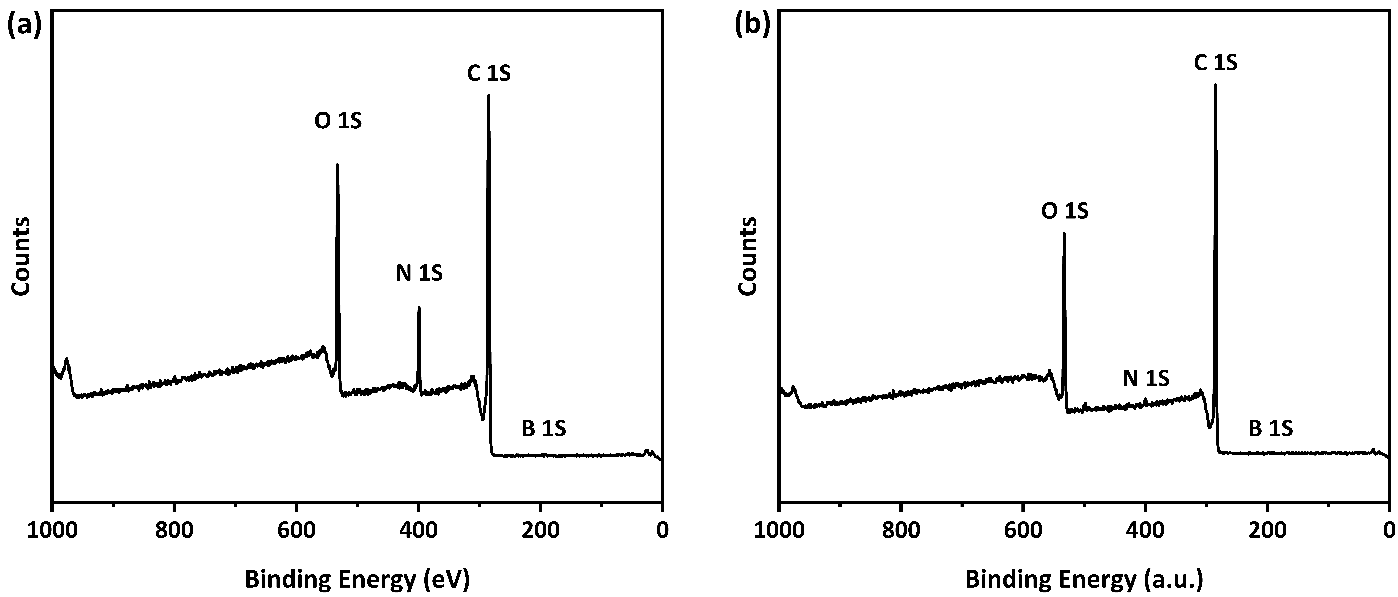
**

Figure S2 XPS survey for (a) G-CQDs and (b) Y-CQDs

**
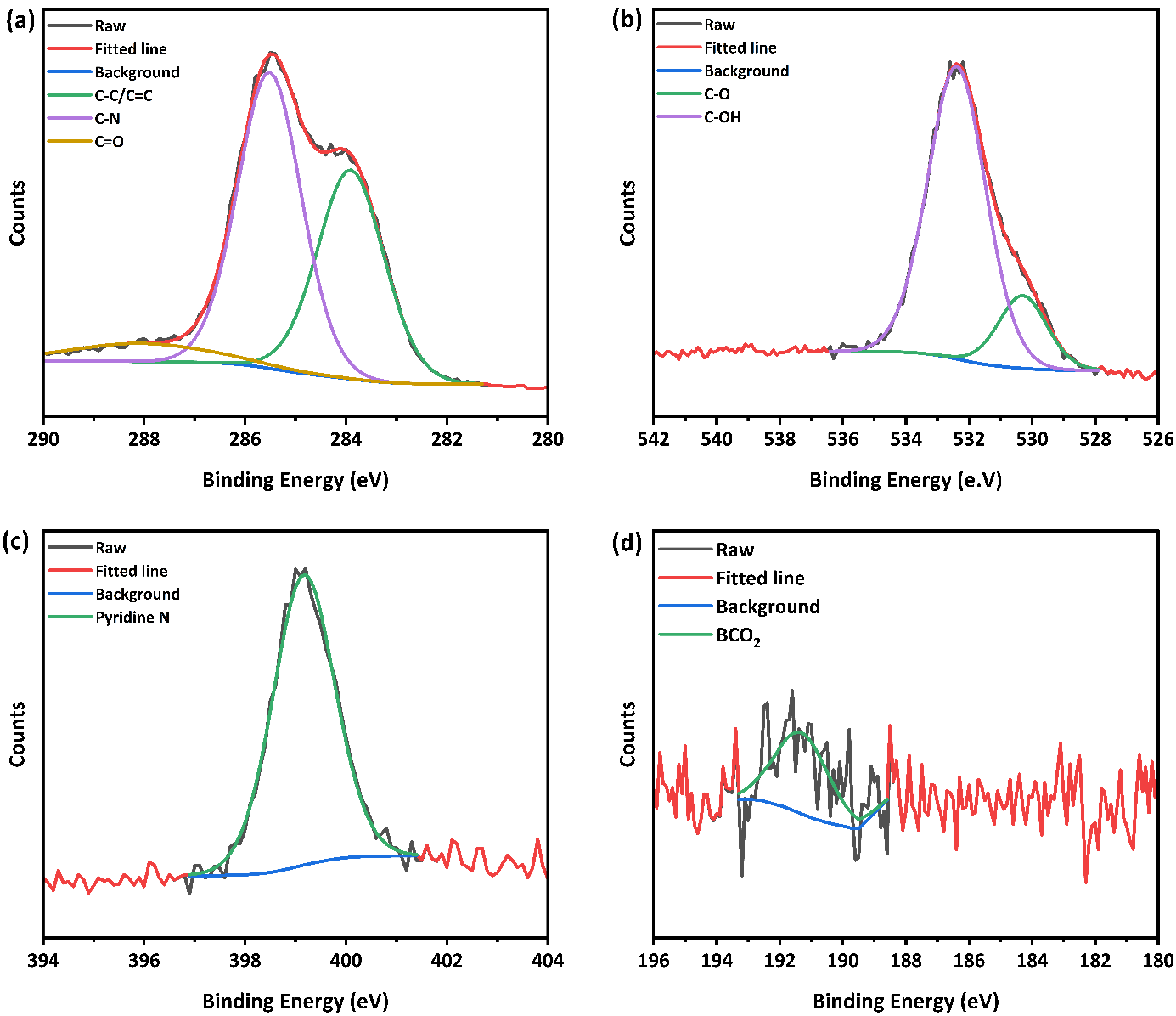
**

Figure S3 High-resolution XPS spectra of G-CQDs. (a) C 1s, (b) O 1s, (c) N 1s, and (d) B 1s

**
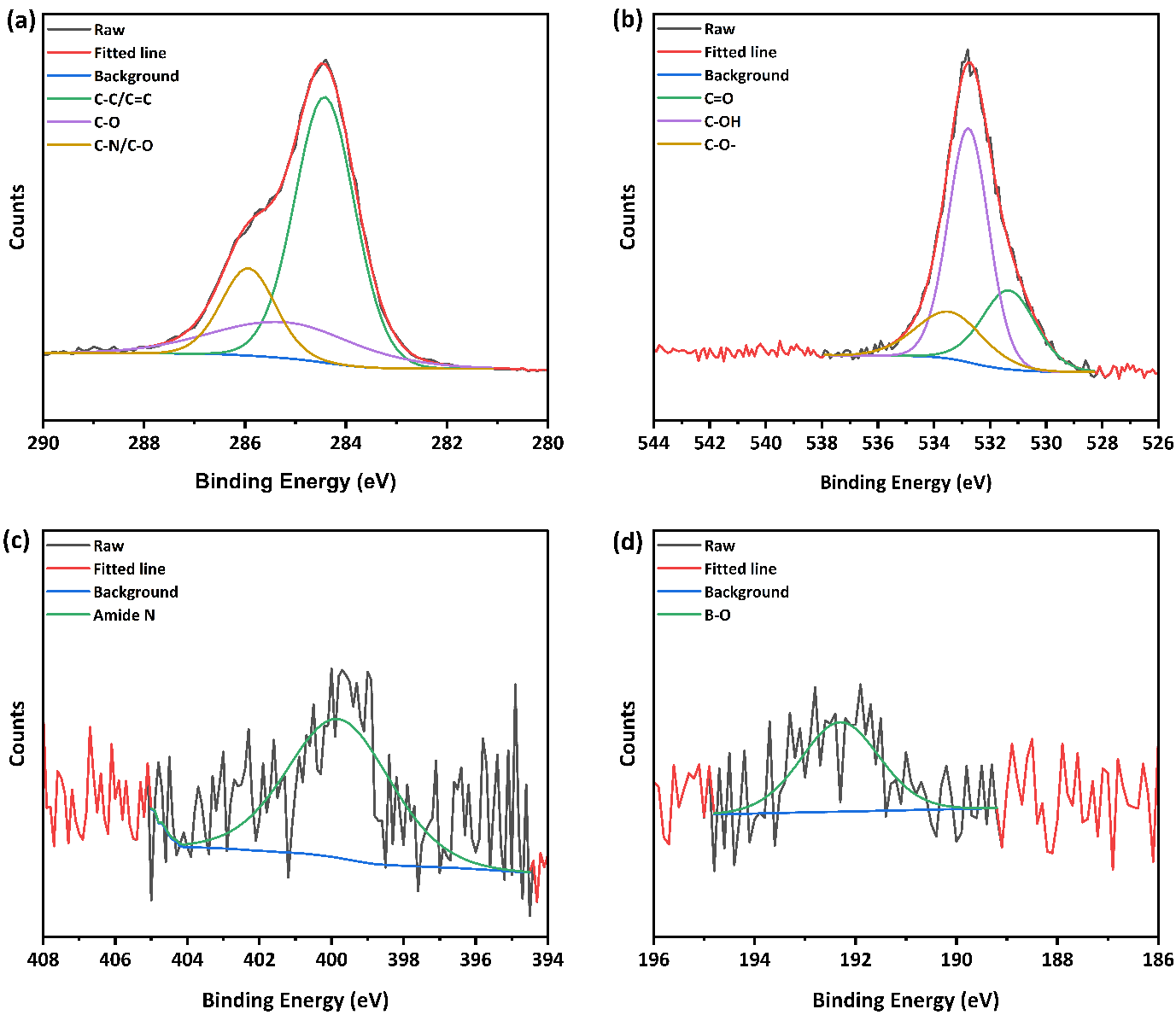
**

Figure S4 High-resolution XPS spectra of Y-CQDs. (a) C 1s, (b) O 1s, (c) N 1s, and (d) B 1s

**
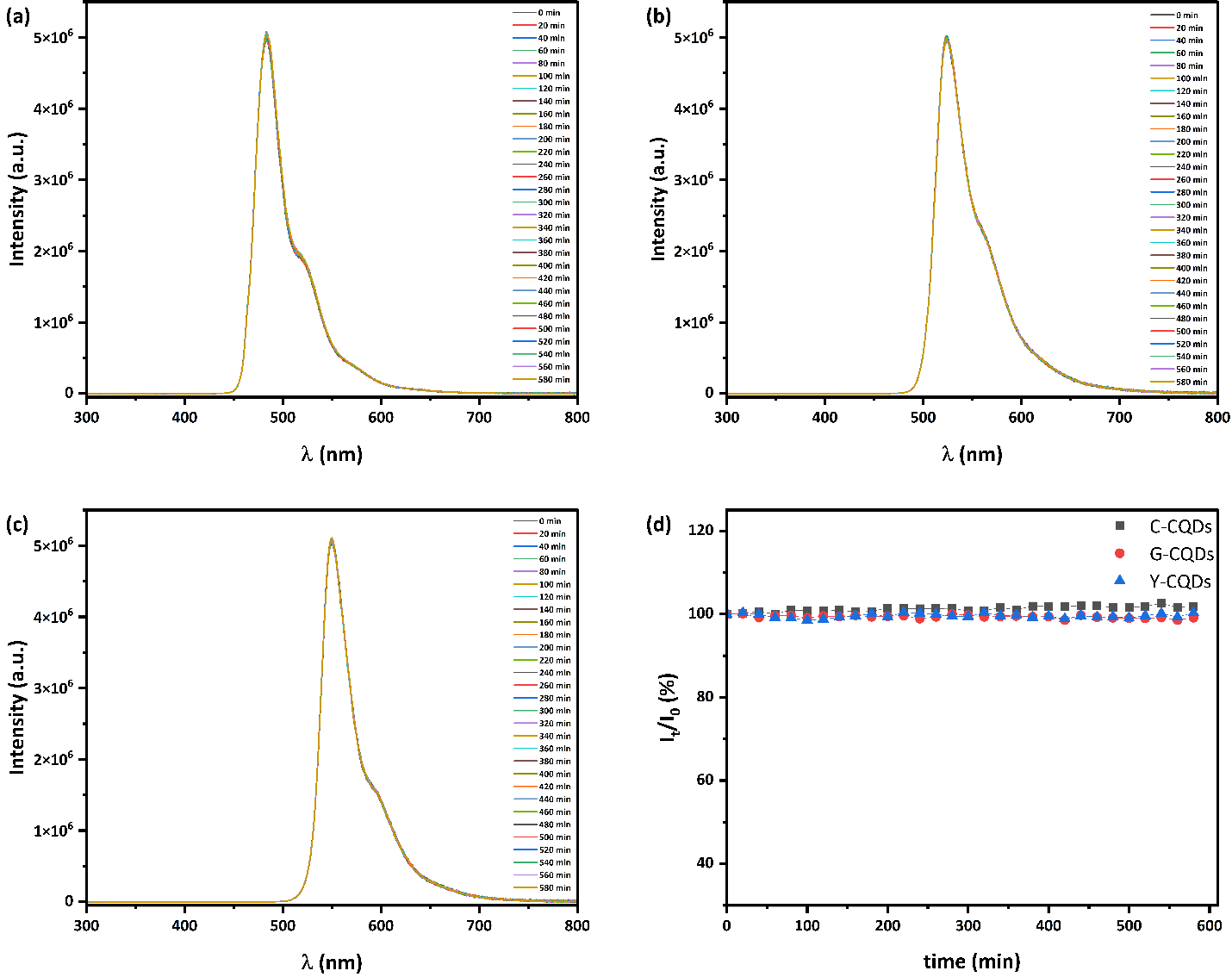
**

Figure S5 PL stability of (a) C-CQDs, (b) G-CQDs, (c) Y-CQDs, and (d) relative emissions for C-, G-, Y-CQDs over time

Table S1 comparison of the optical properties of the synthesized multicolor CQDs and other CQDs reported in literature.

|  | Excitation (nm) | Emission (nm) | Size (nm) | Quantum Yield (%) | Ref |
| --- | --- | --- | --- | --- | --- |
| M-CQDs  O-CQDs | 500  450 | 605  611 | ---  5.6 | ---  16.7 | 1 |
| N-doped CQDs | 420 | 475 | 2.4 | 51.4 | 2 |
| b-CQDs  yg-CQDs  R-CQDs | 355  440  600 | 450  540  665 | 1.71  1.95  2.42 | 25  72  47 | 3 |
| G-CQDs | 410 | 521 | 3.8 | 48.8 | 4 |
| C-CQDs  G-CQDs  Y-CQDs | 462  505  535 | 482  527  560 | 1.7  2.6  3.8 | 65  50  55 | This work |


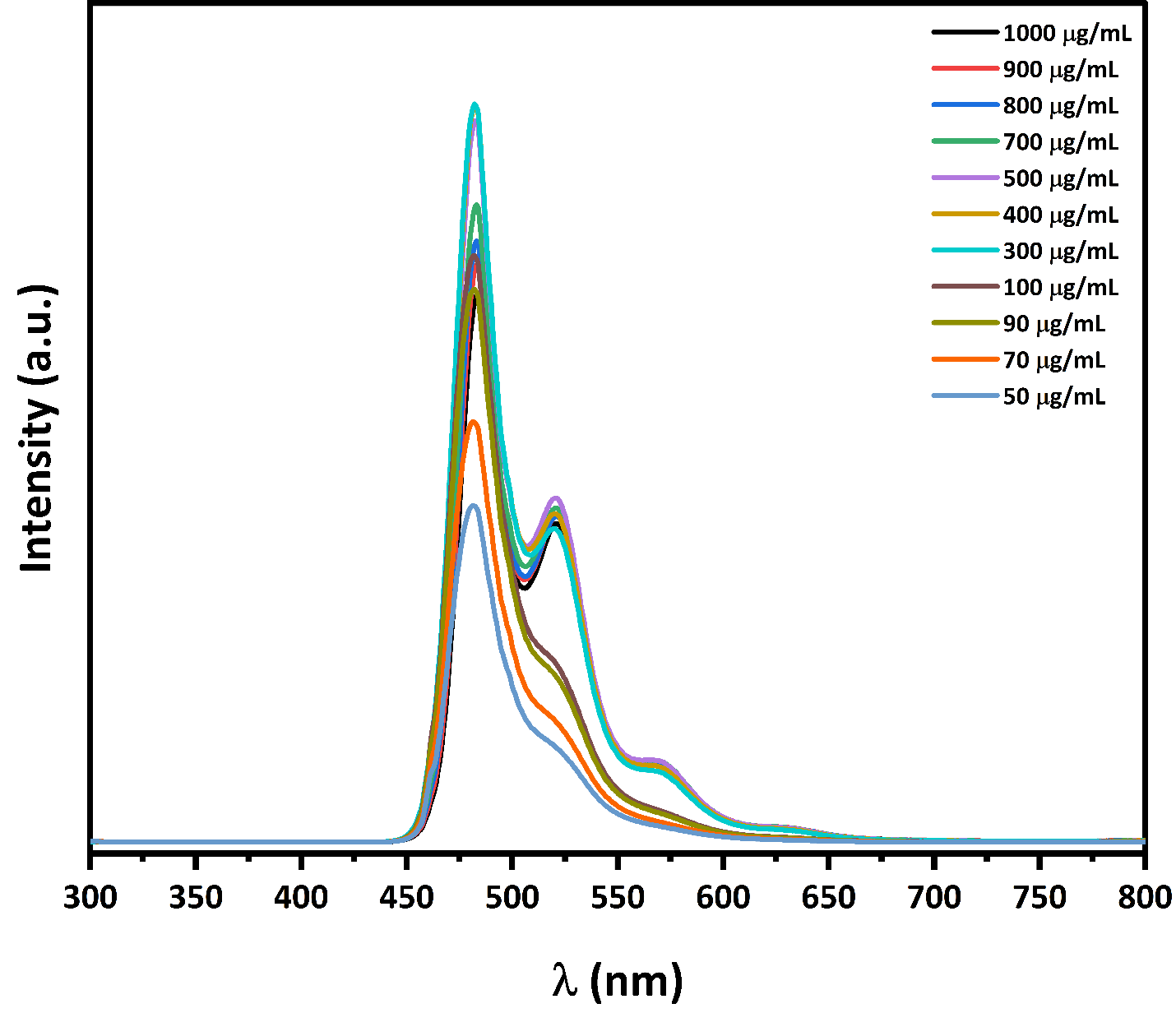


Figure S6 PL emission of C-CQDs at different concentrations.


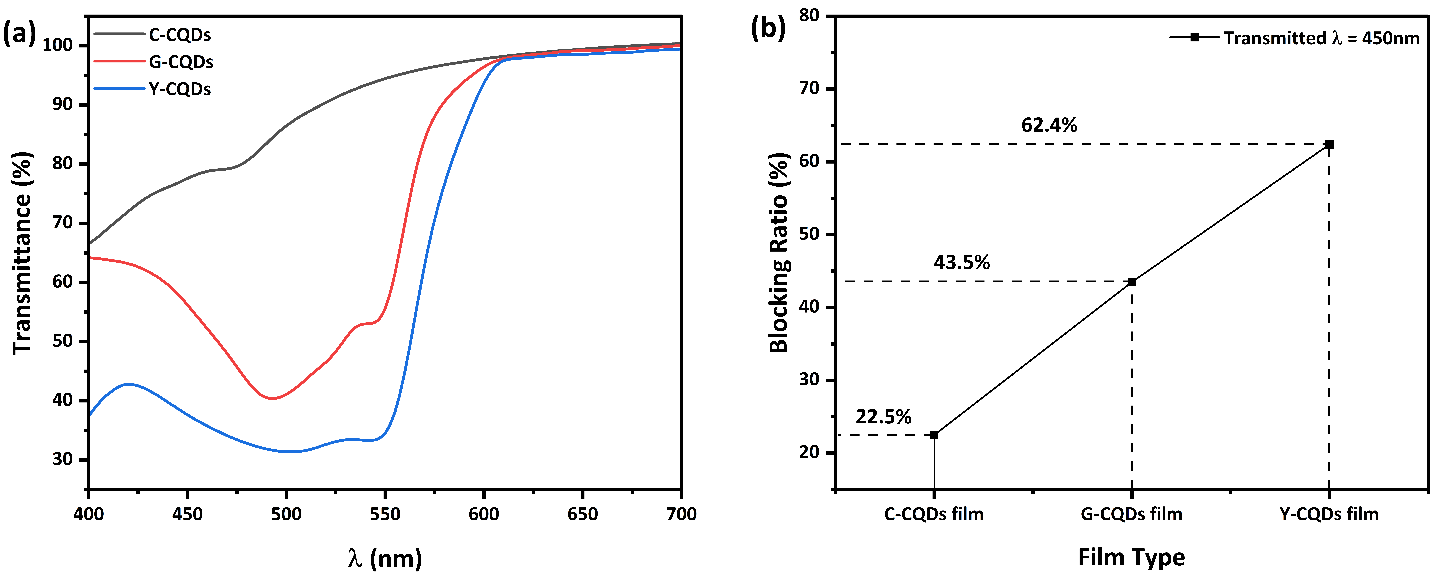


Figure S7 (a) transmission spectra of the fabricated multicolor CQDs films, (b) Blue blocking ratio of same CQDs films

References

1. Kumari, R. & Kumar Sahu, S. Synthesis of Longer-Wavelength-Emissive Carbon Quantum Dots for WLEDs and Investigation of Their Photoluminescence Properties. *ChemistrySelect* **3**, 12998–13005 (2018).

2. Feng, X. T. *et al.* Luminescent carbon quantum dots with high quantum yield as a single white converter for white light emitting diodes. *Appl Phys Lett*, **107**, (2015).

3. Wang, L. *et al.* *Full-Color Fluorescent Carbon Quantum Dots*. *Sci. Adv*, **6**, (2020).

4. Qiu, H. *et al.* Green-light-emitting carbon dots via eco-friendly route and their potential in ferric-ion detection and WLEDs. *Mater Adv* **3**, 7339–7347 (2022).
